# Editorial: Hepatocyte nuclear factor 4 alpha – new insights into an old receptor

**DOI:** 10.3389/fendo.2024.1491965

**Published:** 2024-09-25

**Authors:** Frances M. Sladek, Udayan Apte, Poonamjot Deol

**Affiliations:** ^1^ Department of Molecular, Cell and Systems Biology, University of California, Riverside, Riverside, CA, United States; ^2^ Department of Pharmacology, Toxicology and Therapeutics, University of Kansas Medical Center, Kansas City, KS, United States; ^3^ Department of Microbiology & Plant Pathology, University of California, Riverside, Riverside, CA, United States

**Keywords:** HNF4alpha, HNF4gamma, nuclear receptor, liver, intestines, metabolism, transcription factor, diabetes

**Figure 1:**
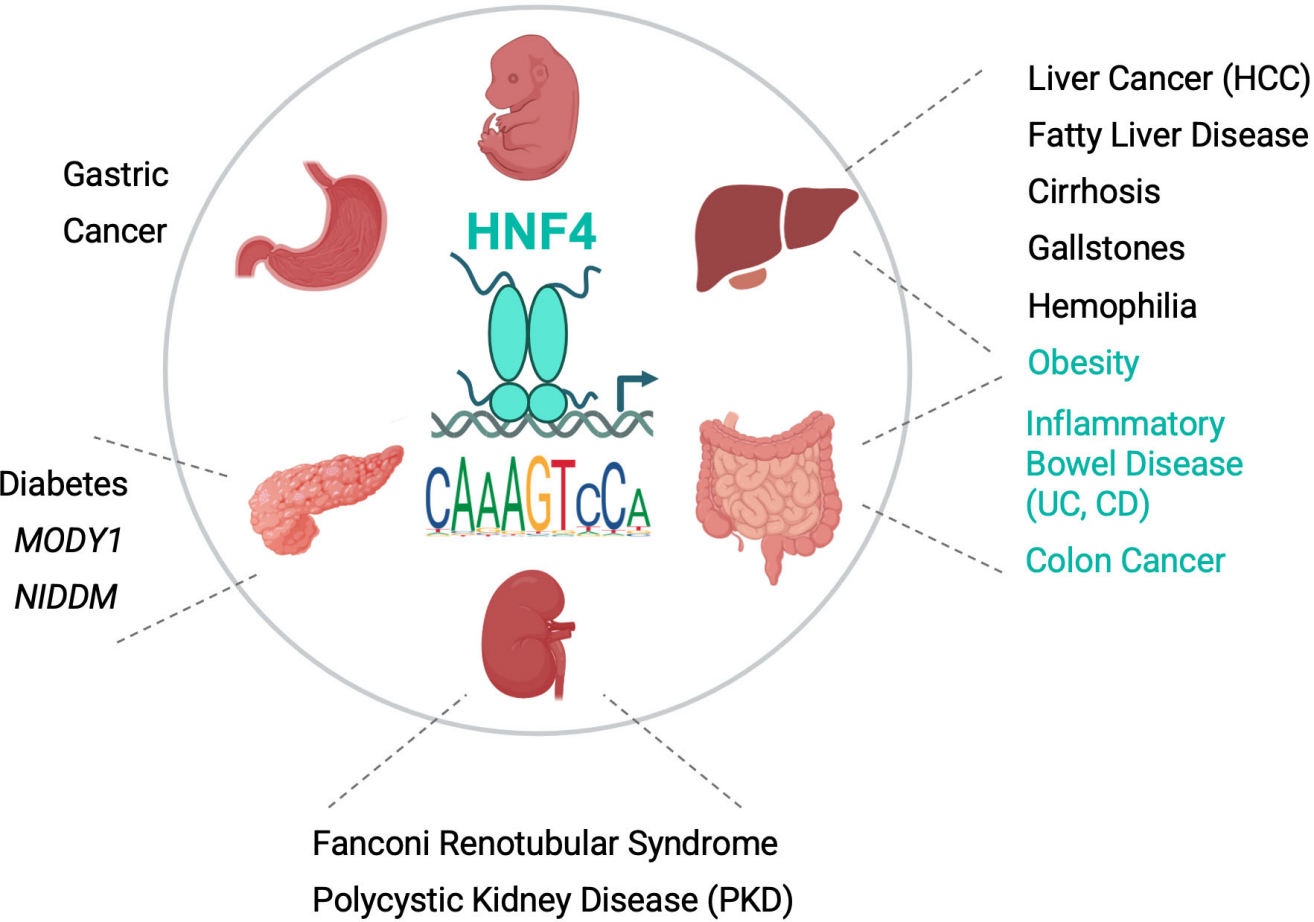
Role of HNF4 in physiology and disease. Schematic of an HNF4 homodimer bound to a promoter and a sequence logo of a canonical HNF4 DNA binding site from JASPAR. The different sized N- and C-termini reflect alternative splicing in HNF4α. Organs in which HNF4α and HNF4γ are expressed are indicated along with associated diseases due to: mutations in the *HNF4A* gene (e.g., MODY1) and HNF4α DNA binding sites in target genes (e.g., *F9* in Hemophilia); association with human SNP variants (e.g., Gallstones, Obesity, UC, CD); and/or dysregulation in human tissues (e.g., Liver, Colon and Gastric Cancer). Black, diseases associated with HNF4α; Teal, diseases associated with both HNF4α and HNF4γ. HCC, hepatocellular cancer; UC, Ulcerative Colitis; CD, Crohn’s Disease; NIDDM, Non Insulin-Dependent Diabetes Mellitus. Image created with BioRender.com. See Vemuri et al. for levels of expression of *HNF4A* and *HNF4G* in different tissues and articles in this Research Topic and (8, 9), for references for diseases.

Hepatocyte nuclear factor 4 alpha (HNF4α), cloned over 30 years ago based on its ability to bind a couple of DNA elements in liver-specific genes, is now considered to be the master regulator of liver-specific transcription. HNF4α is also expressed in several other tissues, including intestines, kidney, stomach and pancreas, and is linked to several human diseases (**Figure 1**). As a member of the nuclear receptor (NR) superfamily of ligand-dependent transcription factors, HNF4α has two highly conserved domains for DNA binding (DBD) and ligand binding (LBD) and binds DNA as a homodimer. This Research Topic contains four reviews and three original research articles covering some of the most important areas of research on HNF4α and its paralog HNF4γ.

The review by Beinsteiner et al. highlights the evolutionary origins of HNF4α as one of two original members of the NR family which, along with RXR, have been found in the most primitive metazoans on Earth. They map several mutations in *HNF4A* found in diabetes, including Maturity Onset Diabetes of the Young 1 (MODY1), as well as numerous phosphorylation, acetylation, ubiquitination and sumoylation sites onto the 3D structure of the full length protein. In an original research article Zhang et al. identify three new mutations in *HNF4A* causing MODY1 in the Chinese population, bringing the total number of mutations mapped in the human *HNF4A* gene to well over 100, the vast majority of which are related to MODY1. The patients are heterozygous for the mutations, as are all the other MODY1 patients, demonstrating the essential nature of HNF4α.

The review by Rastinejad summarizes the search for the endogenous HNF4α ligand as well as the efforts to crystallize the DBD plus LBD structure of HNF4α, the first such structure for any of the 48 human NRs. While fatty acids clearly bind HNF4α (and HNF4γ), their role as classical NR ligands remains ambiguous. What is clear is that the 3D structure of HNF4α contains a ‘convergence zone’ between the DBD and LBD that allows for allosteric interactions such that signals originating from one domain can influence a distant domain.

The review by Radi et al. summarizes the long history of HNF4α including its role in the developing embryo and adult liver. A highly conserved two-promoter structure (P1 and P2) drives the expression of multiple transcript variants in the liver and other tissues. The authors describe an exon-swap mouse model that allows for an *in vivo* analysis of the roles of the transcripts generated from the different promoters: a useful model given that the whole body knockout is an embryonic lethal. The mechanisms underlying the switch between the two promoters, including an anti-sense transcript, a non coding RNA and DNA methylation, are discussed.

The original research article by Deans et al. uses the exon swap mice to elucidate the role of P2- versus P1-HNF4α isoforms in the adult liver. P2-HNF4α was originally identified in an embryonic cancer cell line but is now known to be expressed in the normal adult liver during periods of fasting, high fat diet and alcohol-associated liver disease, as well as liver cancer. The authors use multiple ‘omics approaches to show not only that HNF4α is one of the most highly expressed transcription factors in the adult liver but also that the hundreds of genes uniquely regulated by P2-HNF4α (including several cytochrome P450 genes) are consistent with a role in metabolism. They show that the differential gene expression is likely due to interactions with co-regulators rather than alterations in DNA or chromatin binding and propose a potential role for the HNF4α isoforms in the circadian switch between carbohydrate and lipid metabolism.

The review by Vemuri et al. focusses on the intestines which express both P1- and P2-HNF4α as well as HNF4γ. HNF4α binds DNA as a homodimer and cannot heterodimerize with other NRs, with the exception of HNF4γ. Like HNF4α, HNF4γ has been shown to bind fatty acids although, as with HNF4α, the role of ligand binding in receptor function is not clear. Both HNF4 genes play a key role in intestinal maturation, differentiation and regeneration as well as stem cell renewal (via fatty acid oxidation) and barrier function, allowing for one paralog to compensate for the loss of the other. HNF4α intersects with the immune system in multiple ways and, along with HNF4γ, protects against a chronic inflammatory state in the gut. Both HNF4 genes are implicated in the intestinal entry of SARS-CoV2 via regulation of the *Ace2* and *Tmprss2* genes in the intestinal epithelium. Interestingly, three distinct high fat diets have also been shown to alter the expression of these (and other) COVID-related genes as well as *Hnf4a* in the mouse intestines (1).

The original article by Kotulkar et al. explores the interaction between HNF4α and the proto-oncogene c-Myc in liver regeneration after partial hepatectomy. Deletion of HNF4α increases the expression of c-Myc and cyclin D1 (*Ccnd1*) while the double knockout of HNF4α and c-Myc decreases hepatocyte proliferation demonstrating that HNF4α is critical for both termination of liver regeneration and survival after partial hepatectomy. Another intriguing observation is the emergence of HNF4α+ hepatocytes in HNF4α knockout mice, presumably from the cholangiocytes. This work highlights a role for HNF4α in regulating cell proliferation, as well as basic metabolism.

Critical areas of future HNF4 research include the role of: i) HNF4α in the intersection between cell metabolism and proliferation; ii) the different splice variants and the factors that regulate splicing as well as the alternate *HNF4A* promoters; iii) post translational modifications (PMTs) in HNF4 and how they can be impacted by SNPs in the human population (2); iv) HNF4α versus HNF4γ in the intestines (and other tissues), including homo- versus heterodimers and their interaction with the microbiome (3); v) the incredible diversity of HNF4 and other NR binding motifs and how those might be impacted by SNPs (4); vi) the function of HNF4 in tissues outside of the liver and intestines and interorgan crosstalk with non expressing tissues such as adipose (5, 6); vii) ligand binding and identification of additional ligands, including potential therapeutic drugs; and viii) HNF4 in xenobiotic and drug metabolism (7). Finally, given the ancient origins of HNF4, it will be important to continue exploring new ways in which this ‘old’ receptor acts in all animal organisms.
